# Supplementary material for: Assessment of Condylar Changes in Patients with Degenerative Joint Disease of the TMJ After Stabilizing Splint Therapy: A Retrospective CBCT Study
Source: Diagnostics (Basel). 2024 Oct 19;14(20):2331. doi: 10.3390/diagnostics14202331 (PMC11507354; doi:10.3390/diagnostics14202331)
Supplement: Supplementary file 1 [file diagnostics-14-02331-s001.zip › Supplementary Table S1.pdf]

Supplementary Table S1. Study subject's demographics information.

Age (year)	T2-T1 Duration (Month)	Sex		Race
		F	M	
42.90 ± 18.34	16.98 ± 2.52	18	4	Caucasian
